# Porcine Reproductive and Respiratory Syndrome Virus Modulates the Switch of Macrophage Polarization from M1 to M2 by Upregulating MoDC-Released sCD83

**DOI:** 10.3390/v15030773

**Published:** 2023-03-17

**Authors:** Xingyu Gong, Tianyi Ma, Qiaoya Zhang, Yanhong Wang, Chengchuang Song, Min Lai, Chunlei Zhang, Xingtang Fang, Xi Chen

**Affiliations:** 1 Institute of Cellular and Molecular Biology, School of Life Science, Jiangsu Normal University, Xuzhou 221116, China; 2 College of Veterinary Medicine, Qingdao Agricultural University, Qingdao 266000, China

**Keywords:** PRRSV, macrophage polarization, sCD83, MoDCs

## Abstract

Porcine reproductive and respiratory syndrome virus (PRRSV), the most economically important infectious disease of pigs, elicits poor innate and adaptive immune responses. Soluble CD83 (sCD83), a secretion from various immune cell populations, especially MoDCs, is involved in negatively regulating the immune response. We speculate sCD83 may be a critical factor in the process of PRRSV-coordinated macrophage polarization. In this study, we found that PAMs co-cultured with PRRSV-infected MoDCs inhibited the M1 macrophage while enhancing the M2 macrophage. This was accompanied by a decrease in the pro-inflammatory cytokine TNF-α and iNOS and an increase in the anti-inflammatory cytokine IL-10 and Arg1. Meanwhile, sCD83 incubation causes the same specific effects lead to a switch in macrophage from M1 to M2. Neutralization of sCD83 removes the inhibitory effects of PRRSV on PAMs. Using reverse genetics, we generated recombinant PRRSVs with mutations in N protein, nsp1α, and nsp10 (knockout sCD83-concerned key amino acid site). Four mutant viruses lost the suppression of M1 macrophage markers, in contrast to the restriction of the upregulation of M2 macrophage markers. These findings suggest that PRRSV modulates the switch of macrophage polarization from M1 to M2 by upregulating the MoDC-induced secretion of CD83, providing new insights into the mechanism by which PRRSV regulates host immunity.

## 1. Introduction

Porcine reproductive and respiratory syndrome (PRRS), resulting from the porcine reproductive and respiratory syndrome virus (PRRSV), is one of the most important swine diseases threatening pigs’ health at all ages and causing substantial economic losses worldwide [1]. PRRSV is a positive-strand RNA virus belonging to the Arteriviridae family, whose genome has nine open reading frames and encodes seven structural proteins and 16 non-structural proteins [2,3]. During PRRSV infection, every viral protein could be associated with the immunomodulation capability of the virus. A typical characteristic of PRRSV infection in pigs is evading host defenses through the suppression of innate immunity [3,4]. The absence of a classical innate antiviral immune response occurs as a result of multiple strategies, such as influencing the production of inflammatory and immunomodulatory cytokines, suppressing or enhancing interferon production, enhancing immunosuppressive cytokine expression, or disturbing the development of immune cells including monocytes, macrophages, and T/B cells [1,4,5,6,7,8,9,10,11,12].

PRRSV infection modulates the ability of MoDCs to stimulate the proliferation of T cells via the induction of sCD83 production [13,14,15]. These functions result from the amino acids R43 and K44 in the N protein, the P192-196 and G214-216 domains in nsp10 and the L5, D6, G45, G48, L61, P62, R63, F65, and P66 domains in nsp1α. The N protein of the PRRSV strain BB0907 is involved in IL-10 induction, which stimulates the development of Tregs and weakens T cell proliferation in the host [13,14]. PRRSV infection increases the expression of CXCL-10 and CXCR3 in porcine lung injury, and the CXCL-10/CXCR3 in macrophage may mediate tissue repair by regulating the macrophage expression of Arg1, VEGFa, and TNF-α [16]. PRRSV could restrain the expression of SLA-I, SLA-II, CD80, and CD40, thus impairing the normal antigen presentation of MoDCs and leading to an inadequate adaptive immune response. Furthermore, PRRSV also enhanced Th2-type cytokine IL-10 secretion and reduced Th1-type cytokine IL-12p40, IFN-γ, and TNF-α secretion, resulting in a Th1/Th2 imbalance [17]. Recombinant PRRSV-2 antigen A1 can stimulate the repolarization of M2 PAMs to M1, and hence offer broad protection for both PRRSV-1 and PRRSV-2 strains of infection, further stimulating the Th1 response, along with the activation of T cells and the secretion of IFN-γ from T cells, respectively [18].

Macrophages play an immunity role in modulating the innate immune response, which can be differentiated into two classical groups: pro-inflammatory M1 and alternative anti-inflammatory M2 phenotypes that differ in their inflammatory cytokines/chemokines, surface receptor expression and ability to modulate pathogen clearance [19,20,21,22,23,24,25,26,27]. The M1-activated macrophages secrete a mixture of pro-inflammatory cytokines (TNF-α, IL-6, and IL-12), inducing a high production of nitric oxide synthase (iNOS) involved in the elimination of pathogens [19,23]. In contrast, M2 alternatively activated macrophages produce anti-inflammatory cytokines (IL-10 and TGF-β), express arginase 1 (Arg1), inhibit the inflammatory reaction, and easily cause immunotolerance [23,24]. Accumulating evidence in recent years demonstrates that most viral infections could impact macrophage polarization between the M1 and M2 phenotypes, and this is one of the crucial mechanisms used by viruses to evade host immune responses [28,29,30,31,32,33,34,35]. Foot and mouth disease virus (FMDV) upregulates TNF-α, IL-12, Type I IFN, and ISG including viperin in a time-dependent manner and induces phenotypic and functional M1 polarization [28]. Swine influenza virus (SIV) infection modulates a switch in macrophage polarization from M1 to M2 through the Aflatoxin B1 [29]. Influenza A virus (IAV) H1N1 infection seems to sequentially activate the M1 polarization and recruitment of macrophages via an autophagy–exosome-dependent pathway [30]. HBV infection can promote the expression of B7x, which in turn regulates the Th17/Treg balance and affects the expression of HBsAg and HBeAg. The mechanism used by B7x likely involves the promotion of macrophage polarization and apoptosis [31].

CD83 is a marker of mature dendritic cells (DCs), which can exist in two forms: membrane CD83 (mCD83) and soluble CD83 (sCD83). mCD83, produced by monocyte-derived DCs (MoDCs), monocytes, B lymphocytes, and myelocytes, appears to lead to stimulated or regulatory functions in various immune cell populations [14,36,37,38,39,40,41]. Moreover, mCD83 can be released into the microenvironment to become soluble CD83, which displays an immunosuppressive function in the immune response, such as inhibiting DC-mediated T cell stimulation and interfering with the maturation of DCs and the cellular cytoskeleton [36,42,43].

sCD83 contributes to immune homeostasis by enhancing the responsiveness of antigen-specific Th2 cell activities via suppression of Bcl2L12 expression [41]. Human cytomegalovirus (HCMV) and Human T-cell leukemia virus type I (HTLV-I) infection increase the shedding of mCD83 in human DCs; the resulting sCD83 suppresses allogeneic T cell proliferation and survival [44]. In our previous study, we found that PRRSV infection upregulates CD83 expression, resulting in the shedding of sCD83 from MoDCs. PRRSV inhibits the expression of the MHC-I complex proteins TAP1 and ERp57 while disturbing MoDC-mediated T-cell proliferation [13,14]. Viral proteins were examined individually to reveal that N, nsp1α, and nsp10 regulate the CD83 promoter. By constructing targeted mutations, we found that the amino acids R43 and K44 of the N protein, amino acids L5, D6, G45, G48, L61, P62, R63, F65, and P66 of Nsp1α, as well as P192 and G214 of nsp10, play important roles in inducing CD83 production [13,14,15]. However, the underlying linkage among these factors (PRRSV, macrophage, and sCD83) is yet to be elucidated.

In this study, we investigated the possible correlation among PRRSV, M1/M2 polarization, and sCD83 in PAMs. We found that infection with the wild-type PRRSV BB0907 strain targets the expression of sCD83, thus regulating macrophage polarization from the M1 phenotype to M2 phenotype bias. These results might provide new theories and insights into the immune mechanism underlying immune suppression by PRRSV infection, with implications for the development of vaccine optimization strategies.

## 2. Materials and Methods

### 2.1. Cells and Viruses

Marc-145 cells, derived from African green monkey kidney cells, are highly permissive to PRRSV infection. HEK 293T cells (a human embryonic kidney cell line) and MARC-145 cells were obtained from the ATCC and held in our laboratory. The cells were incubated in Dulbecco’s modified essential medium (DMEM; Gibco, Carlsbad, CA, USA) and supplemented with 10% heat-inactivated fetal bovine serum (FBS; Gibco) containing 100 U/mL penicillin and 100 μg/mL streptomycin at 37 °C with 5% CO_2_. Primary alveolar macrophages (PAMs) were collected via bronchoalveolar lavage from six-week-old PRRSV-negative pigs, and all processes of the animal were authorized and supervised by the rules approved by the State Council of the People’s Republic of China for experimental animal care and use. PAMs were cultured in an RPMI1640 medium supplemented with 10% fetal bovine serum (FBS).

HP-PRRSV isolate BB0907 (GenBank accession number HQ315835) was passaged 10 times through Marc-145 cells and is designated as BB in this study. The virus was isolated in 2009 in Guangxi Province, China, and has been maintained as a stock in our laboratory. Classical PRRSV (C-PRRSV) strain S1 (GenBank accession number AF090173) was isolated from pigs with clinical signs of PRRS in Jiangsu Province in 1997. The low pathogenicity PRRSV (LP-PRRSV) isolate NT0801 (GenBank accession number HQ315836) was isolated in Jiangsu Province, China, in 2008. Recombinant viruses [rN-43-2A (mutant sites at residues R43 and K44 of N protein), rnsp10-2m (mutations at rP192-5A and rG214-3A of nsp10), rnsp1α-3m (includes mutations in rL5-2A, rG45A/G48A, and rL61-6A), and rN/nsp10 (includes mutations in R43 and K44 of N protein and rP192-5A and rG214-3A of nsp10)] were rescued from infectious clone pCMV-BB0907 (constructed in our laboratory) and were propagated in Marc-145 cells upon recovery. The viral titers were determined using the Reed–Muench method and expressed as tissue culture infective dose 50% (TCID_50_).

### 2.2. Western Blot

Levels of iNOS, Arg1, N protein, and GAPDH were measured by Western blotting. Briefly, treated cells were collected and lysed on ice for 30 min in a protein isolation buffer, subjected to SDS-PAGE, and then transferred to a polyvinylidene difluoride (PVDF) membrane (Millipore, Billerica, MA, USA). The membrane was blocked with 5% low-fat milk at 37 °C for 1 h and then probed with MAb anti-N (1:100; prepared in the College of Veterinary Medicine, Nanjing Agricultural University, Nanjing, China), rabbit anti-iNOS (1:2000, Proteintech), rabbit anti-Arg1 (1:1000; ProteinTech, Rosemont, IL, USA), and rabbit anti-GAPDH (1:1000; Beyotime Biotechnology, Shanghai, China) at 37 °C for 2 h. After washing, the membrane was incubated with HRP-conjugated anti-mouse or anti-rabbit secondary antibody (1:10,000, ABclonal). Bound proteins were detected by enhanced chemiluminescence (Biosharp, Hefei, China) and imaged using a chemiluminescence imaging system (Tanon, Shanghai, China).

### 2.3. Measurements of TNF-α, IL-10 and sCD83

PAM supernatants were collected for the measurement of TNF-α, IL-10, and sCD83. TNF-α and IL-10 were measured by a radioimmunoassay using a Porcine IL-10 ELISA Kit and Porcine TNF-α ELISA kit (Beyotime Biotechnology, Shanghai, China). sCD83 were measured by radioimmunoassay using a Human sCD83 ELISA Kit (Abcam, Cambridge, UK) according to the manufacturer’s instructions. The optical density (OD) was measured with a microplate reader at 450 nm, and the secreted protein concentrations were calculated according to the standard curve.

### 2.4. RT-qPCR

Quantitative RT-PCR was performed to measure iNOS and Arg1. Total RNA from PAMs was extracted using a Qiagen RNeasy kit (Qiagen, Hilden, Germany). cDNA was synthesized using an RT-PCR kit (TaKaRa, Kusatsu, Japan) according to the manufacturer’s instructions. Quantitative real-time PCR was performed using SYBR Premix Ex TaqTM II (Tli RNaseH Plus) (TaKaRa). Relative quantification of target gene expression was calculated using the 2^−ΔΔCq^ method. Data are presented as fold changes in gene expression, normalized to GAPDH and relative to the mock-infected control. Each reaction was performed in triplicate, and the data are expressed as means ± standard errors of the means (SEM). Primer sequences are shown in Table 1.

### 2.5. Construction of Infectious PRRSV cDNA Clones

**Figure 1:**
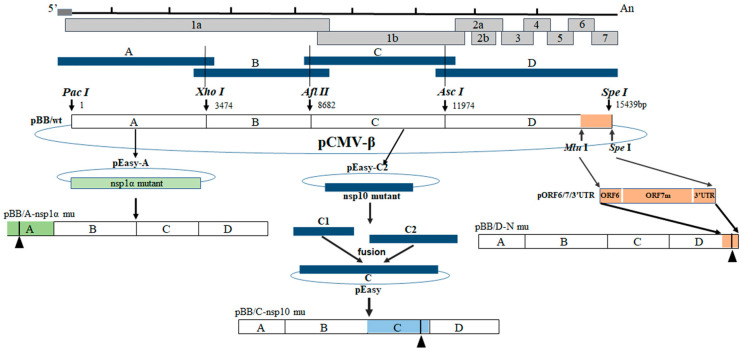
Construction strategy for full-length cDNA clones for the mutant N, nap1α, and nsp10 of PRRSV.

The full-length PRRSV genome was amplified using the five primer pairs listed in Table 2. A recombinant plasmid (pCMV-BB) containing a full-length cDNA copy of the virus was constructed, as shown in Figure 1 and designated as pCMV-BB [13,14,45,46]. It was used as the backbone to construct N protein nsp1α and nsp10 mutants in a PRRSV BB0907 background. Mutagenesis of the N protein was performed on the D fragment of pCMV-BB. Mutagenesis of the nsp1α protein was performed on the A fragment of pCMV-BB. Mutagenesis of the nsp10 protein was performed on the C fragment of pCMV-BB. Fragments containing mutations in N protein, nsp1α, and nsp10 were obtained by site-directed PCR mutagenesis using pBB/wt as the template. The PCR products were inserted into a pEASY-Blunt Simple vector (designated pEASY-Am, pEASY-Cm, pEASY-Dm). The plasmids were then digested with PacI/XhoI, Afl II/Asc I, or Asc I/Spe I, and fragments containing the mutation were ligated into the same restriction enzyme-digested pBB/wt to obtain the full-length mutant cDNA clones pBB/N-R43/K44A, pBB/nsp10 (P192-5A/G212-5A), pBB/nsp1α-3m, and pBB/N/Nsp10, respectively (Figure 1). MARC-145 cells were transfected with each plasmid to generate the mutant viruses rBB/wt, rN-43-2A, rnsp10-2m, rnsp1α-3m, and rN/Nsp10. All mutant PRRSV strains were repaired in MARC-145 cells. Briefly, plasmids containing the mutations were modified to restore the N, nsp1α, or nsp10 wild-type genotypes. Fragments containing the repaired mutations were ligated into digested pBB/wt. All recombinant viruses and amino acid substitution primers are listed in Table 3. MARC-145 cells were transfected with the resulting full-length cDNA clones to generate repaired PRRSV. Transfections were conducted with Lipofectamine 3000 reagent according to the manufacturer’s protocol. The supernatants were harvested and serially passaged four times in MARC-145 cells until about 80% of cells exhibited cytopathic effects (CPEs). Passage 2 to passage 5 virus stocks were prepared in the same manner. All full-length mutant clones were verified by nucleotide sequencing (data not shown). The plasmids were isolated using the QIAprep spin miniprep kit (Qiagen).

### 2.6. One-Step Viral Growth Curves

MARC-145 cells, seeded in 24-well plates, were inoculated with 10^6^ TCID_50_ of the virus. At 6, 12, 24, 36, 48, and 72 h post-infection, 100 μL of the infected cell supernatant was removed and the same volume of fresh medium was added back to each well. All samples were stored at −70 °C until virus titration. Virus titers were determined as TCID_50_.

### 2.7. Statistical Analysis

Data were expressed as the mean ± SEM of at least three independent experiments for each cellular experimental group. The data were evaluated by one-way analysis of variance (ANOVA), followed by post-hoc Tukey’s test using GraphPad Prism version 5.0 (GraphPad Software, San Diego, CA, USA). *p* values of less than 0.05 (* *p* < 0.05, ** *p* < 0.01, *** *p* < 0.001) were considered statistically significant.

## 3. Results

### 3.1. Alterations in M1 and M2 Macrophage Types after PRRSV Infection In Vitro

**Figure 2:**
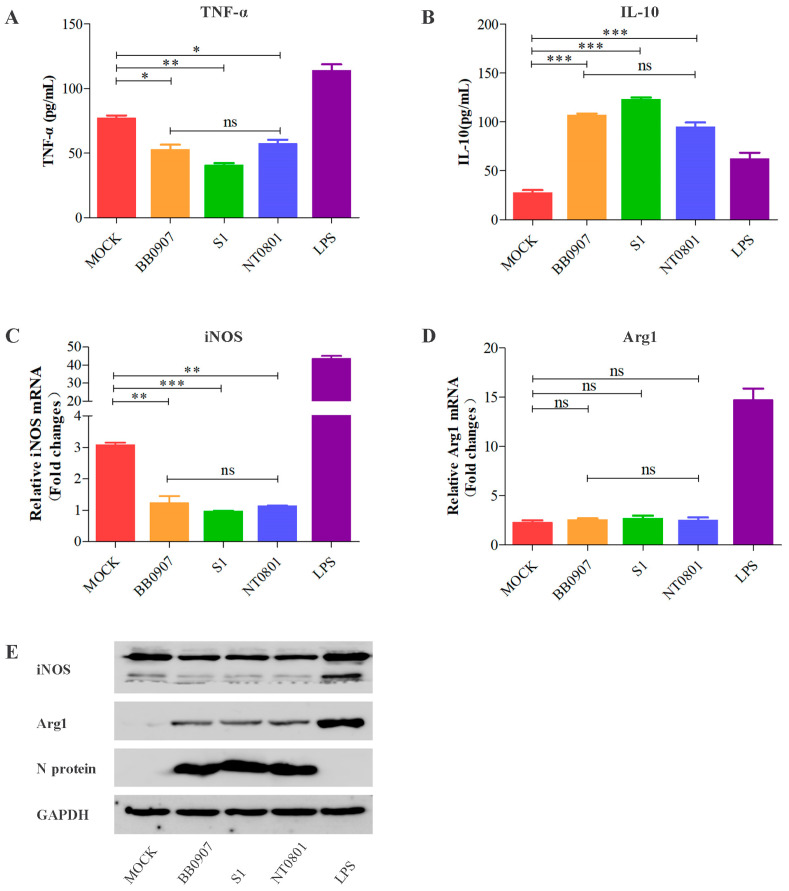
PAMs differentiation into M1 or M2 macrophages is impaired by PRRSV infection. HP-PRRSV BB0907, C-PRRSV S1, and LP-PRRSV NT0801 used at an MOI of 1. Mock-infected and LPS-treated MoDCs were used as negative and positive controls, respectively. Cells were then co-cultured with PAMs. After 24 h, (**A**) TNF-α production of M1 macrophages was significantly lower, while (**B**) IL-10 production of M2 macrophages was obviously higher in PRRSV-infected PAMs than negative controls. (**C**,**D**) The mRNA expression level of iNOS by M1 macrophages and Arg1 by M2 macrophages were impacted in PRRSV-infected PAMs. (**E**) The protein level of iNOS of M1 macrophages and Arg1 of M2 macrophages in PAMs infected by PRRSV were analyzed by Western blot. All assays were repeated at least three times, with each experiment performed in triplicate. Bars represent means ± SEM from three independent experiments. ***, *p* < 0.001; **, *p* < 0.01; *, *p* < 0.05; ns, not significant.

MoDCs were incubated with strains of different virulence levels (HP-PRRSV BB0907, classical PRRSV [C-PRRSV] S1, and low pathogenicity PRRSV [LP-PRRSV] NT0801) for 36 h, and cell supernatant was co-cultured with PAMs. After 24 h, PAMs were polarized to M1 and M2 macrophages. Production of the cytokines TNF-α and IL-10, reflective of the pro-inflammatory and anti-inflammatory function of M1 and M2 macrophages, respectively, was assessed using ELISA (Figure 2A,B). The mRNA and protein expression levels of iNOS (a marker of M1) and Arg1 (a marker of M2) were detected by RT-qPCR (Figure 2C,D) and Western blot (Figure 2E). As expected, TNF-α release and iNOS expression of M1 macrophages from PRRSV-infected groups were significantly lower than that of the control group. However, changes in the mRNA level of Arg1 were less marked, while Arg1 protein expression and IL-10 release of M2 macrophages from PRRSV-infected cells were obviously higher. Taken together, these data indicate that PRRSV infection induces a switch in alveolar macrophage polarization from M1 to M2. Because the HP-PRRSV infection has caused immense economic damage in China during the pandemic, the highly virulent HP-PRRSV BB0907 strain was selected for subsequent experiments.

### 3.2. PRRSV Regulates Macrophage Polarization in a Dose-Dependent Manner

**Figure 3:**
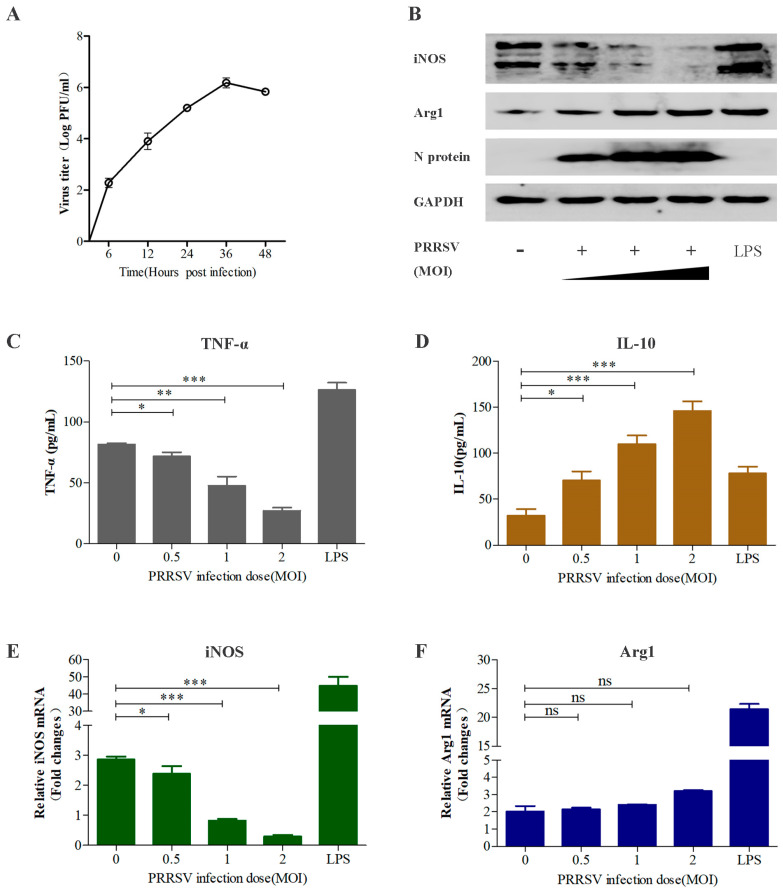
PRRSV influences macrophage polarization in a dose-dependent manner. (**A**) PRRSV infection kinetics were measured in the supernatants of infected MoDCs by TCID50 assay. MoDCs were infected with PRRSV at an MOI of 0, 0.5, 1, and 2 for 36 h, (**B**) the protein level of iNOS by M1 macrophages and Arg1 by M2 macrophages was measured by Western Blot. (**C**,**D**) The levels of TNF-α and IL-10 release were detected by ELISA. (**E**,**F**) The mRNA level of iNOS by M1 macrophages and Arg1 by M2 macrophages were obtained by RT-qPCR. Data are presented as the means ± SEM (*n* = 3). ***, *p* < 0.001; **, *p* < 0.01; *, *p* < 0.05; ns, not significant.

Firstly, PRRSV titers in infected MoDCs peaked at 36 hpi (Figure 3A); accordingly, we choose PRRSV to infect MoDCs for 36 h in subsequent trials. To optimize viral infection, MoDCs were incubated with PRRSV at an MOI of 0, 0.5, 1, or 2 for 36 h, Samples were collected and added to PAMs. Cells treated with LPS were used as a positive control. As shown in Figure 3B,C,E, the level of TNF-α release, iNOS mRNA and protein expression were significantly lower in cells infected with PRRSV at an MOI of 2 than in those infected at an MOI of 0.5. Otherwise, the levels of IL-10 release and Arg1 protein expression were prominently higher at an MOI of 2 than those infected at an MOI of 0.5 (Figure 3B,D). Curiously, the increase of the Arg1 mRNA level was almost negligible in PRRSV infection from MOI 0.5 to 2 (Figure 3F). These results indicate that PRRSV decreases TNF-α release and iNOS expression and increases IL-10 release and Arg1 expression in a dose-dependent manner.

### 3.3. sCD83 Modulates a Switch in Macrophage Polarization from M1 to M2

**Figure 4:**
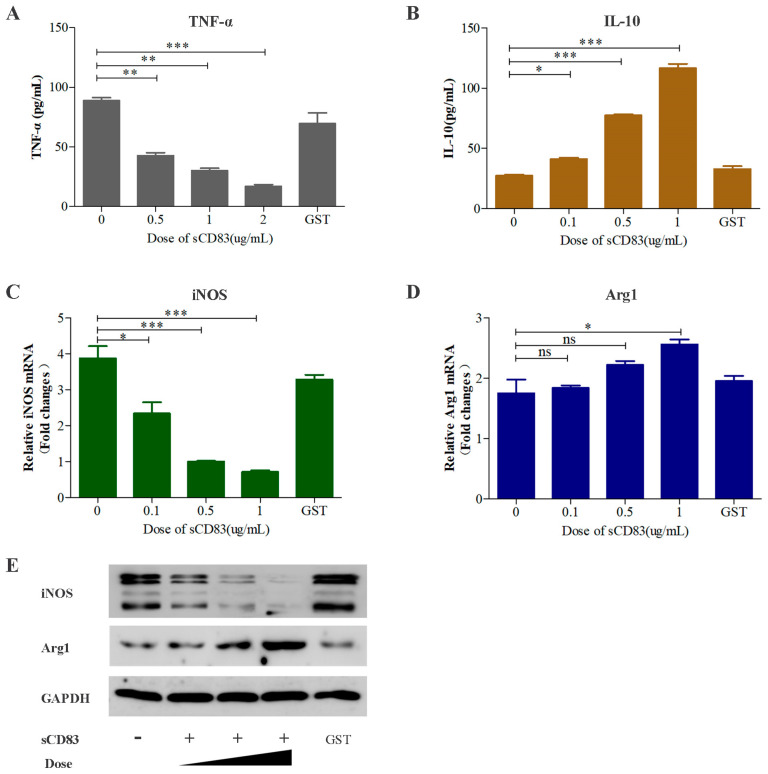
Recombinant sCD83 suppresses the expression of M1 macrophage markers but enhances the expression of M2 macrophage markers. Effect of sCD83 protein on M1 and M2 macrophage marker expression in PAMs. PAMs (1.0 × 10^6^) were incubated with 5 μg/mL GST-Cap and 0.1, 1, and 5 μg/mL of sCD83 protein for 36 h. (**A**,**B**) Cell supernatant was examined by porcine TNF-α ELISA Kit and porcine IL-10 ELISA Kit. iNOS (**C**) and Arg1 (**D**) mRNA expression were analyzed by RT-qPCR. mRNA levels were calculated relative to known amounts of a template and normalized to GAPDH expression. (**E**) Cell lysates were examined by Western blotting with anti-iNOS and anti-Arg1 antibodies. PBS treatment was used as a negative control and endogenous GAPDH expression was used as an internal control. Results are representative of three independent experiments. Data are represented as means ± SEM. ***, *p* < 0.001; **, *p* < 0.01; *, *p* < 0.05; ns, not significant.

To further investigate the effects of sCD83 on the phenotypic switch in macrophage polarization, PAMs, in the presence of GST-sCD83 protein at concentrations of 0.1, 0.5, and 1 μg/mL, were measured by Western blotting, ELISA, and RT-qPCR. GST-Cap (5 μg/mL) treatment was used as a control group. As shown in Figure 4A,C,E, levels of TNF-α release and iNOS expression were reduced, but the levels of IL-10 release and Arg1 expression were enhanced in cells treated with GST-sCD83 in a dose-dependent manner (Figure 4B,E). Simultaneously, the sCD83-induced mRNA level of Arg1 hardly budged (Figure 4D). The results of the sCD83 treatment corresponded well with the effects of PRRSV infection.

### 3.4. Anti-CD83 Restores a Switch in Alveolar Macrophage Polarization from M1 to M2 in PRRSV Infection

**Figure 5:**
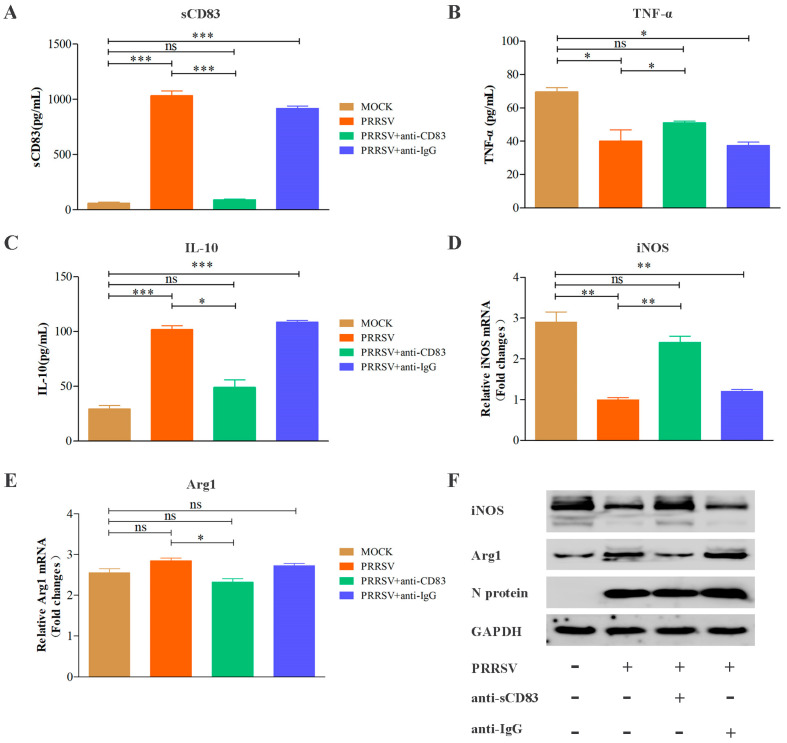
Anti-CD83 antibody blocks the ability of PRRSV to induce a switch in macrophage polarization from M1 to M2. MoDCs were pre-treated with rabbit anti-CD83 antibody to remove sCD83, or with isotype (rabbit IgG) antibody as a negative control in the cell culture medium. MoDCs were then infected with PRRSV at an MOI of 1 for 36 h, and supernatants from these cultures were added to PAMs. Cell supernatants were studied for sCD83 (**A**), TNF-α (**B**), and IL-10 (**C**) secretion. Cell lysates were analyzed for mRNA and protein levels of iNOS (**D**), Arg1 (**E**), and N proteins (**F**) using RT-qPCR and Western blotting (**F**), respectively. GAPDH was used as a loading control. Data are representative of at least three independent experiments. ***, *p* < 0.001; **, *p* < 0.01; *, *p* < 0.05 compared with the mock treatment group.

To test if macrophage polarization responds to sCD83 level in PRRSV infection, MoDCs were incubated with rabbit anti-CD83 or rabbit IgG at 10 μg/mL for 1 h and then infected with PRRSV at an MOI of 1. After 36 h, cells were co-cultured with PAMs. The level of sCD83 in MoDCs supernatant was tested by ELISA (Figure 5A). Figure 5B,D,F show that the levels of TNF-α release and iNOS expression were obviously higher in the anti-CD83-treated group. Similarly, cells pre-treated with anti-CD83 led to lower levels of IL-10 release and Arg1 expression than other groups (Figure 5C,E,F). This result demonstrates that immunodepletion of soluble CD83 largely restores the macrophage polarization from M1 to M2 resulting from PRRSV infection.

### 3.5. Construction and Identification of Mutant Recombinant PRRSV

**Figure 6:**
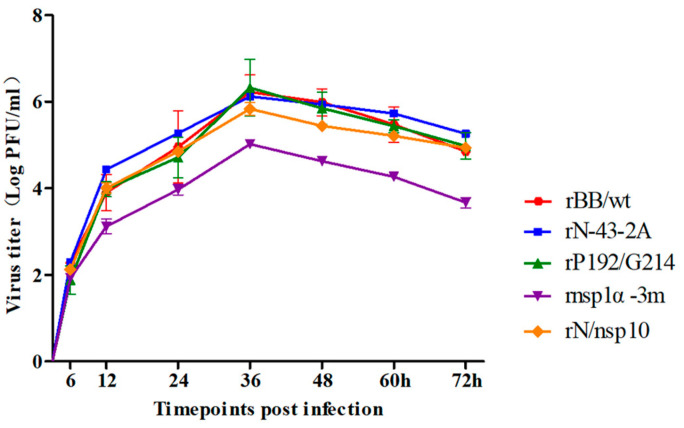
Identification of sCD83-relative amino acid mutant PRRSV. Multi-step growth kinetics of PRRSV in MARC-145 cells after infection by the indicated viruses at an MOI of 0.1. Culture supernatant was collected at the indicated time points for viral titration. Results are expressed as 50% tissue culture infective dose (TCID_50_). Titers from three independent experiments are shown as means ± SEM (error bars).

To confirm the identity of the amino acid residues responsible for CD83 promoter activation, viruses containing mutations in N, nsp10, and nsp1α were constructed using infectious PRRSV cDNA clones (Figure 1). Four viruses were successfully rescued. These were rN-43-2A (mutant sites at residues R43 and K44 of N protein), rnsp10-2m (mutations at rP192-5A and rG214-3A of nsp10), rnsp1α-3m (mutations in rL5-2A, rG45A/G48A, and rL61-6A) and rN/nsp10 (mutations in R43 and K44 of N protein and rP192-5A and rG214-3A of nsp10). The viruses were functional, as judged by the presence of a cytopathic effect 4 days post-transfection. In addition, rN-43-2A, rnsp10-2m, and rN/nsp10 exhibited growth kinetics similar to those of the parental wild-type rBB/wt virus (Figure 6), but rnsp1α-3m grew more slowly than wild-type rBB/wt infection, resulting in an approximately 10-fold difference in titer.

### 3.6. Effect of Mutant Viruses on Macrophage Polarization from M1 to M2 by sCD83

**Figure 7:**
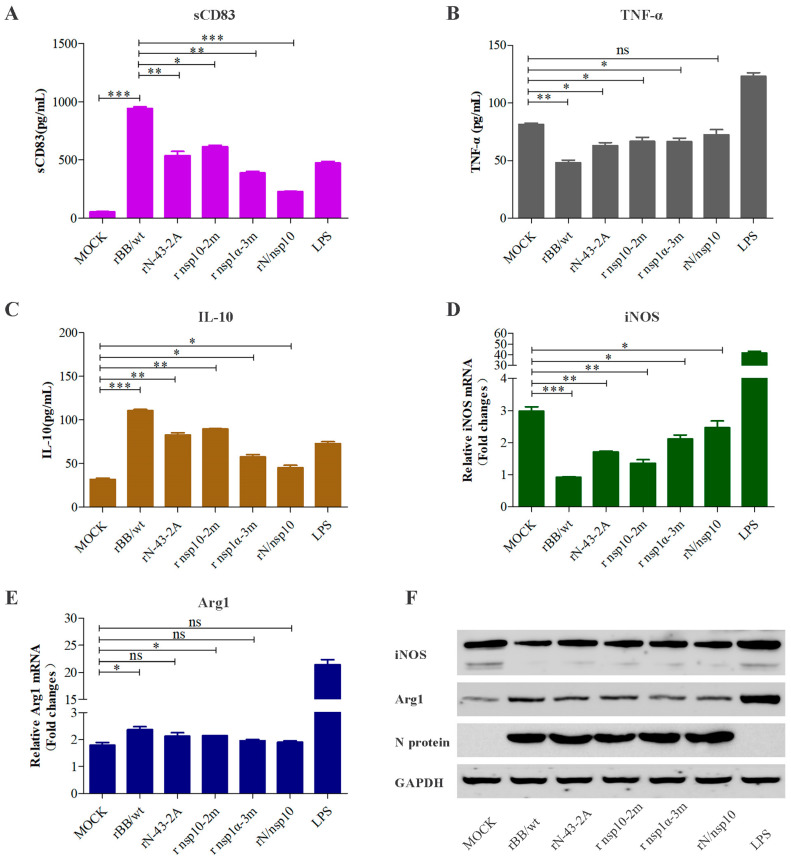
All mutations impair the ability of PRRSV to impact macrophage polarization from M1 to M2. MoDCs were mock infected or infected with recombinant PRRSV [rBB/wt, rN-43-2A, rnsp10-2m, rnsp1α-3m, and rN/nsp10] at an MOI of 1. After incubation for 36 h, supernatants from these cultures were added to PAMs for 24 h. The release of sCD83 (**A**), TNF-α (**B**), and IL-10 (**C**) was detected by a specific ELISA Kit. (**D**,**E**) iNOS and Arg1 mRNA levels were determined by qPCR. (**F**) Cell lysates were examined by Western blotting with anti-iNOS and anti-Arg1. Replication of PRRSV was analyzed by Western blotting using anti-N protein. GAPDH was used as a loading control. Data are representative of three experiments as means ± SEM. ***, *p* < 0.001; **, *p* < 0.01; *, *p* < 0.05.

To determine whether mutant viruses affect macrophage polarization, MoDCs were infected with the four mutant viruses at an MOI of 1. Twenty-four hours after infection, cells were co-cultured with PAMs and analyzed by ELISA, RT-qPCR, and Western blot. As shown in Figure 7A, the mutants decreased their ability to secrete sCD83 compared with the parental virus rBB/wt. Levels of TNF-α release and iNOS protein expression were higher in untreated cells infected by rN-43-2A, rnsp10-2m, rnsp1α-3m, and rN/nsp10 than cells infected by wild-type rBB/wt (Figure 7B,F). In contrast, the four mutants significantly decreased in levels of IL-10 release and Arg1 protein expression relative to those in the rBB/wt group (Figure 7C,F). Similarly, the mRNA level of iNOS is consistent with the result of the Western blot, but the Arg1 mRNA level barely changed in each mutant viral group (Figure 7D,E).

## 4. Discussion

Macrophages were identified as phagocytic cells, having a well-defined role in the host’s response including eliminating pathogens, inflammation, metabolic diseases, autoimmune diseases, and acute lung injury [22,23,24,25,26,27,29,30]. Macrophages commonly change into two forms: the classically activated (M1) macrophages (inflammatory monocytes) or the alternatively activated (M2) macrophages (anti-inflammatory monocytes). In different pathophysiological conditions and surrounding microenvironments, macrophages phenotypically switch between M1 and M2, i.e., macrophage polarization [23,24]. The Th1 cytokine IFN-γ and the ligand of TLR4, lipopolysaccharide (LPS), polarize monocytes toward classically activated (M1) macrophages and possess antiviral activity [24]. M2 statuses represent cell activation statuses polarized by interleukin (IL)-4/IL-13, which are secreted by Th2 cells and related to immune tolerance [23]. Consequentially, M1 phenotype macrophages express higher levels of pro-inflammatory mediators including granulocyte-macrophage colony-stimulating factor (GM-CSF), TNF-α, IL-1, IL-6, and IL-12 and preferentially express inducible nitricoxide synthase (iNOS; NOS2) [32]. Conversely, M2 macrophages produce abundant anti-inflammatory cytokines, such as IL-10, and exhibit a high expression of arginase 1 (Arg-1) [33]. Cumulative research has shown that macrophage polarization is required for many crucial processes of viral infection. COVID-19 could induce STAT-3 expression and then contribute to the development of M2-like macrophages, which could regulate viral replication [47]. HIV pronounced and prolonged the shift from the M1 to M2 macrophages program and elevated the level of IL-10, thus contributing to the failure of all immunological functions and clinical collapse [48]. The New World arenavirus Junin (JUNV) P strain induced higher levels of IRF-1, SOCS1, and SOCS3, triggering macrophage activation and eliciting a more anti-inflammatory M2 response, which may allow JUNV to evade immune detection [49]. HCV core protein interacts with TLR2/STATs signaling, leading to dysfunction of monocyte polarization toward both M1 and M2 macrophages, while intervening on macrophage-induced autologous and allogeneic CD4+ T cell activation [35]. Indeed, several recent studies have demonstrated that macrophages are polarized to M1/M2 type within the innate immune system, which is associated with viral infections and antiviral states.

Porcine reproductive and respiratory syndrome virus (PRRSV), a virulent pathogen of swine, suppresses the innate immune response and induces persistent infection [5,50,51]. More detailed reviews of host interactions with PRRSV conclude that most PRRSV strains delayed and impaired the adaptive immune response, inhibiting the release of pro-inflammatory cytokines, promoting anti-inflammatory cytokines, and modulating the activity and function of immune cells, including dendritic cells, macrophages, natural killer cells, T lymphocytes, and B lymphocytes [1,3,5,13,14]. PRRSV infection upregulates the expression of negative immune regulators including NF-k B inhibitors (NFKBIA, NFKBID, NFKBIZ, and TNFAIP3) and T-cell exhaustion markers (programmed death ligand-1 [PD-L1], PD-L2, interleukin-10 [IL-10], IDO1, and transforming growth factor b2 [TGFB2]) in PAMs to modulate the host’s immune response [7]. PRRSV affects the phagocytic capacity of PAM, resulting from interactions with its entry mediators Sn and/or CD163 mediates the inhibitory effect of PRRSV on PAM phagocytosis [52]. PRRSV infection skews macrophage polarization toward the M2 phenotype, and then boosts IFN- and IL-12 secretion and TNF-*a* expression, which is connected with the transformation of A1 (g6Ld10T) and A2 (lipo-M5Nt) antigens [18].

CD83, a glycoprotein member of the Ig superfamily of receptors, usually came in one of two forms: membrane-bound CD83 (mCD83) and soluble CD83 (sCD83) [36]. mCD83 is expressed in a variety of cells, including mature dendritic cells (DCs), activated T cells and T regulatory cells, B cells, and macrophages [53]. Soluble CD83 (sCD83), as an immunosuppressive molecule, is cleaved from membrane-bound CD83 (mCD83) [15,38]. Studies have indicated that sCD83 is involved in negatively regulating the immune response. sCD83 binds directly to MD-2, the TLR4 co-receptor, leading to an induction of the anti-inflammatory mediators IDO and IL-10, inhibiting T cell proliferation, blocking IL-2 secretion, and rendering the downstream functions of T cells [54]. sCD83 modulates monocyte-DC differentiation and negatively influences immune regulation for DC-mediated T cell immune responses [55]. In donor corneal allografts, sCD83 acts on responder cells, including Mφ and DCs, induces tolerogenic APCs (macrophage/monocyte/DCs), decreases allogeneic T cell proliferation, hampers IL-6 and TNF-α secretion, and produces immunoregulatory mediators (IL-10, Ido1, TGF-β, and IL-27), thus leading to improved corneal graft acceptance [56]. sCD83 might play an important role in regulating the immune tolerance in the maternal–fetal interface of pregnant animals by inducing Foxp3þ Treg cell generation, promoting IL-10 and IL-4 expressions, inhibiting the expression of TNF-a and IL-6, and shifting the Th1/Th2 cytokine balance to Th2 dominance [15].

As previously noted, in our research we have found PRRSV stimulates the secretion of CD83 and increases CD83 mRNA levels, leading to a decreased expression of the MHC-peptide complex components TAP1 and ERp57 in MoDCs and inhibiting the ability of MoDCs to stimulate the proliferation of T cells [13]. These effects are derived from key amino acid sites (R43 and K44 in the N protein; L5A, D6A, G45A, G48A, L61A, P62A, R63A, F65A, and P66A in the ZF domain of Nsp1α; and the P192-5 and G214-3 domains in nsp10) in the PRRSV-induced release of sCD83 [13,14]. We speculated that PRRSV might influence the immune response through the microenvironment by regulating macrophages through sCD83 expression. To this end, how PRRSV infection causes the imbalance of macrophage polarization in severe respiratory infections in young pigs and reproductive disorders, and whether this is related to PRRSV-induced sCD83, remains unknown.

Accordingly, our data suggested that PRRSV inhibits the allostimulatory capacity of M1 macrophages but increases the allostimulatory capacity of M2 macrophages. In PAMs, PRRSV infection decreased TNF-α production but increased IL-10 release, as well as induced a high protein level of Arg1 but a low protein level of iNOS. Since sCD83 has an inhibitory effect on immune capacity, including inhibition of DC-mediated T cell stimulation and DC maturation, which can disturb the process of antigen presentation in innate immune cells (such as monocytes, macrophages, and DCs) and modulate the differentiation of immune cells [13,14,15,42]. We further confirmed the function of sCD83 in macrophage polarization. PAMs were exposed to sCD83 (0.5 ug/mL) and our data showed that sCD83 induced a switch in macrophage polarization from M1 to M2 in vitro. Our previous studies have shown that PRRSV infection induces CD83 expression in porcine MoDCs through the NF-κB and Sp1 signaling pathways. Key amino acid sites of viral N, nsp10, and nsp1 can stimulate sCD83 release and bring about immunosuppression in immune cells [13,14]. We further investigated whether PRRSV-induced switches in macrophage polarization from M1 to M2 is mediated through the secretion of sCD83. MoDCs were treated with anti-CD83 (0.5 ug/mL) in advance and then infected with PRRSV. The experimental results show that anti-sCD83 preconditioning restricted the conversion from M1 to M2. Similarly, the iNOS expression and TNF-α secretion were restored markedly, but Arg1 expression and IL-10 secretion was apparently diminished compared to the anti-IgG pre-treated group. Then, four mutant viruses with mutations of the sCD83-relative essential site in the N protein, nsp10, and nsp1 were rescued using cDNA infectious clones. The analysis indicated that all mutant viruses (rN-43-2A, rnsp10-2m, rnsp1α-3m, and rN/nsp10) specifically interfere with functions related to macrophage polarization to different degrees.

In conclusion, this study revealed that monocyte polarization toward both M1 and M2 macrophages is modulated by PRRSV infection through the excretion of sCD83, leading to the dysfunction of M1 macrophages and promotion of M1 macrophages. In PAMs, PRRSV regulates both pro-inflammatory (TNF-α) and anti-inflammatory (IL-10) processes, which contribute to the immune response. We propose that PRRSV-induced sCD83 might offer novel insights into the molecular mechanism of immune suppression by PRRSV.

## 5. Conclusions 

**Figure 8:**
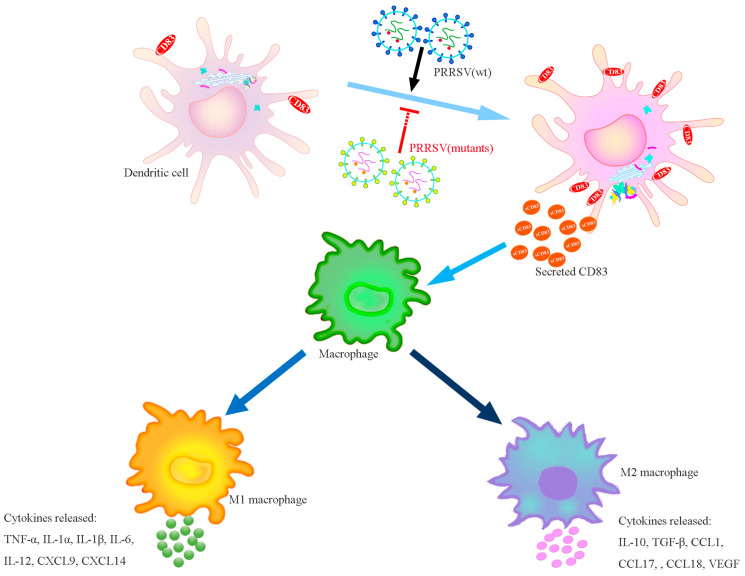
sCD83-mediated influence of macrophage polarization in PRRSV infection.

Infection of MoDCs by PRRSV increases the release of soluble CD83 in particular. sCD83 strongly decreases the TNF-α release and iNOS expression of M1 macrophages but enhances IL-10 release and Arg1 expression of M2 macrophages. Viruses containing mutations in R43 and K44 of the N protein, the domains L5, D6, G45, G48, L61, P62, R63, F65, and P66 of Nsp1α, and the P192-196 and G214-216 domains of nsp10 do not affect sCD83 expression or impact the polarization of the macrophages (Figure 8).

## Figures and Tables

**Table 1:** Sequence of primers used for quantitative real-time PCR.

Name	Sequence (5′-3′)
GAPDH-F	CCTTCCGTGTCCCTACTGCCAAC
GAPDH-R	GACGCCTGCTTCACCACCTTCT
TNF-α-F	ACTCGGAACCTCATGGACAG
TNF-α-R	AGGGGTGAGTCAGTGTGACC
IL-10-F	ACTTTAAGGGTTACCTGGGTTT
IL-10-R	TGCTTCACTTTTGCATCTTC
iNOS-F	GCACCTGCGTTATGCCACCAAC
iNOS-R	TGAGCTGAGCGTTCCAGACCC
Arg-1-F	AGCCCGTGTCAACATGACTTCC
Arg-1-R	TTGTGTTGGCATCTTTACTGA
sCD83-F	CGCTCTCCAGAATGGCTCTT
sCD83-R	ACTCTGCTGTCGTGCAAACT

**Table 2:** Primers used for construction of the infectious cDNA clone of strain BB0907.

Primer	Sequence (5′-3′) ^a^
A-BB0907-Fwd	GCGTTAATTAAACCGTCATGACGTATAGGTGTTG
A-BB0907-Rev	TGTCTCGAGAATCATCTTTGGGAGAAACC
B-BB0907-Fwd	TTCTTAATTAAATGATTCTCGAGACACCGCC
B-BB0907-Rev	GTGCTTAAGTTCATTACCACCTGTAACGGAT
C1-BB0907-Fwd	GCGTTAATTAAAATGAACTTAAGCACCTATGCC
C1-BB0907-Rev	TTGACACAGAGGTAATCGGGTCGCCAGAC
C2-BB0907-Fwd	GTCTGGCGACCCGATTACCTCTGTGTCAA
C2-BB0907-Rev	CGGGGGAAAATGAAACCTCATGCTGGT
D1-BB0907-Fwd	TCGTTAATTAAGTTTCGGGCGCGCCAGAAAGGG
D-BB0907-Rev(SwaI)	TTCGGCTTGGGATTTAAATATGCATTTTTTTTTTTTTTTTTTTTT
D-BB0907-Rev(SpeI)	CTCACTAGTAACGGCCGCCAGTGTGCTGGAATTCGGCTTGGGATTT
XhoI-BB0907-Fwd	GGTACCATGGCCAAACTCGAGGCTTTTGCCGATACC
XhoI-BB0907-Rev	GGTATCGGCAAAAGCCTCGAGTTTGGCCATGGTACC

^a^ The genomic positions for the primers are based on GenBank accession number HQ315835.

**Table 3:** Primers used for construction of the CD83 promoter, N protein, nsp1α, and nsp10 mutant.

Primer	Sequence (5′-3′) ^a^
43ASF	GGCAAGGGACCGGGGGCGAAAAATAGGAAGACA
43ASR	TGTCTTCCTATTTTTCGCCCCCGGTCCCTTGCC
44ASF	AAGGGACCGGGGAAGGCAAATAGGAAGACAAAA
44ASR	TTTTGTCTTCCTATTTGCCTTCCCCGGTCCCTT
P192-5ASF	TGCCGGTTTAACGTTGCAGCAGCTGCAGCGCTGCAATTCCCTGCC
P192-5ASR	GGCAGGGAATTGCAGCGCTGCAGCTGCTGCAACGTTAAACCGGCA
L212-5ASF	CCATGGGTTCGCATCGCGGCCGCCGCTGCGTGTCCTGGCAAGAAC
L212-5ASR	GTTCTTGCCAGGACACGCAGCGGCGGCCGCGATGCGAACCCATGG
L5ASF	GATAAGTCTGGGATAGCTGATCGGTGCACGTGT
L5ASR	ACACGTGCACCGATCAGCTATCCCAGACTTATC
D6ASF	AAGTCTGGGATACTTGCTCGGTGCACGTGTACC
D6ASR	GGTACACGTGCACCGAGCAAGTATCCCAGACTT
G45ASF	CAAGTTCCTGAGCTTGCGGTGCTGGGTCTATT
G45ASR	AAATAGACCCAGCACCGCAGCTCAGGAACTTG
G48ASF	GAGCTTGGGGTGCTGGCTCTATTTTATAGGCCC
G48ASR	GGGCCTATAAAATAGAGCCAGCACCCCAAGCTC
L61ASF	CCACTCCGGTGGACGGCGCCACGTGCATTCCCC
L61ASR	GGGGAATGCACGTGGCGCCGTCCACCGGAGTGG
P62ASF	CTCCGGTGGACGTTGGCACGTGCATTCCCCACT
P62ASR	AGTGGGGAATGCACGTGCCAACGTCCACCGGAG
R63ASF	CGGTGGACGTTGCCAGCTGCATTCCCCACTGTC
R63ASR	GACAGTGGGGAATGCAGCTGGCAACGTCCACCG
F65ASF	ACGTTGCCACGTGCAGCCCCCACTGTCGAGTGC
F65ASR	GCACTCGACAGTGGGGGCTGCACGTGGCAACGT
P66ASF	TTGCCACGTGCATTCGCCACTGTCGAGTGCTCC
P66ASR	GGAGCACTCGACAGTGGCGAATGCACGTGGCAA

^a^ The genomic positions for the primers are based on GenBank accession number XM_001928655.

## Data Availability

All data generated or analyzed during this study are included in this manuscript.
